# Molecular detection and risk factors for *Anaplasma platys* infection in dogs from Egypt

**DOI:** 10.1186/s13071-021-04943-8

**Published:** 2021-08-26

**Authors:** Abdelfattah Selim, Hamdan Almohammed, Abdelhamed Abdelhady, Abdulaziz Alouffi, Fahdah Ayed Alshammari

**Affiliations:** 1 Department of Animal Medicine (Infectious Diseases), Faculty of Veterinary Medicine, Benha University, Toukh, 13736 Egypt; 2 Department of Microbiology and Parasitology, Almaarefa University, Riyadh, 11597 Saudi Arabia; 3 Department of Parasitology and Animal Diseases, National Research Center, Dokki, Giza, Egypt; 4 King Abdulaziz City for Science and Technology, Riyadh, 12354 Saudi Arabia; 5 College of Sciences and Literature Microbiology, Northern Border University, KSA, Arar, Saudi Arabia

**Keywords:** Dogs, *Anaplasma platys*, Conventional PCR, *16S* rRNA gene, Phylogenetic analysis

## Abstract

**Background:**

*Anaplasma platys* is a tick-borne bacterium which infects blood platelets of dogs, causing canine cyclic thrombocytopenia. The disease is distributed worldwide, particularly in the tropics and subtropics, but information on the epidemiology of *A. platys* infection in dogs is fragmentary in many countries, including Egypt. In this study, we investigated the prevalence and risk factors associated with *A. platys* infection in dogs from Egypt.

**Methods:**

A conventional PCR targeting a fragment of the *16S* rRNA gene of *A. platys* was used to screen 500 dogs from five North Egyptian governorates. DNA sequencing and phylogenetic analysis were performed for one of the positive samples.

**Results:**

The overall prevalence of *A. platys* in the studied dogs was 6.4%. Females of the German shepherd breed without veterinary care had higher odds for *A. platys* positivity. High tick infestation and lack of anti-tick treatment were also identified as risk factors for *A. platys* infection. Phylogenetic analysis revealed that the sequence obtained herein was closely related to sequences from Egypt, South Africa and Uruguay.

**Conclusions:**

This is the first large-scale epidemiological study of *A. platys* in Egypt, where female German shepherd dogs without veterinary care, as well as dogs with high tick infestation and without anti-tick treatment are at a higher risk of infection.

**Graphical abstract:**

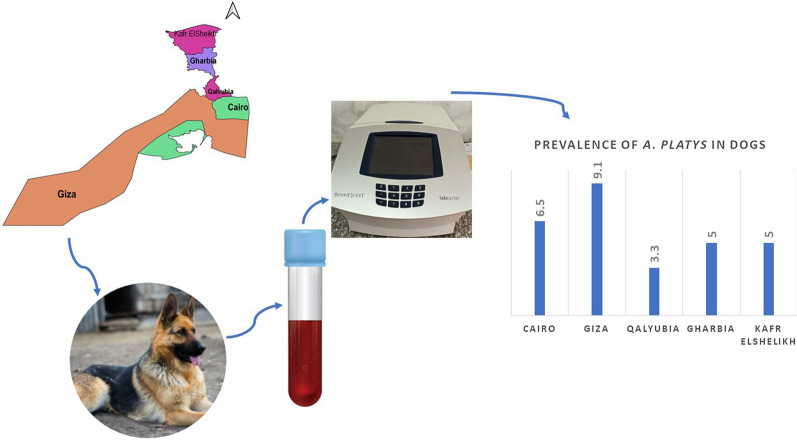

*Anaplasma platys* is a Gram-negative, obligate intracellular bacterium, which is reputed to be transmitted by brown dog ticks *Rhipicephalus sanguineus* sensu lato (s.1.) [1, 2]. *Anaplasma platys* is most commonly found in dogs, but natural infections have also been identified in cats, foxes, wild boars, red deer and a goat [3, 4]. The vast majority of infected dogs are asymptomatic, but bleeding may occur in rare cases, and co-infection with other vector-borne pathogens increases the severity of *A. platys* infection [5].

Molecular and/or serological evidence of *A. platys* in dogs has been reported in countries of different continents, including Europe [6, 7], the Americas [8, 9], Asia [10], Australia [13] and African countries including Kenya, Ivory Coast [14], Tunisia [11], Algeria [12], Morocco [13], Senegal [18], Angola [19] and Sudan [20], among others. More recently, *Anaplasma* spp. was serologically reported in dogs [14] and *A. platys* was molecularly identified in *R. sanguineus* s.l. ticks collected from dogs in Egypt [15]. Moreover, *A. platys*-like variants have been detected in cattle in Menoufia Governorate, Egypt [23]. Nonetheless, information on the epidemiology of *A. platys* in African countries, including Egypt, is still fragmentary. Therefore, the present study aimed to determine the molecular prevalence of *A. platys* infections in dogs from five governorates in northern Egypt and to evaluate the possible risk factors associated with this infection.

The study was conducted in five governorates, including Cairo, Giza, Qalyubia, Gharbia and Kafr ElSheikh, situated in the North of Egypt (Fig. 1). The climatic conditions of these regions are tropical and humid with two distinct seasons: a dry season from May to September and a warm and humid season from October to April. In the dry season, the average temperature ranges between 15 °C and 28 °C; in the rainy season, the annual average rainfall is 200 mm.

**Fig. 1:**
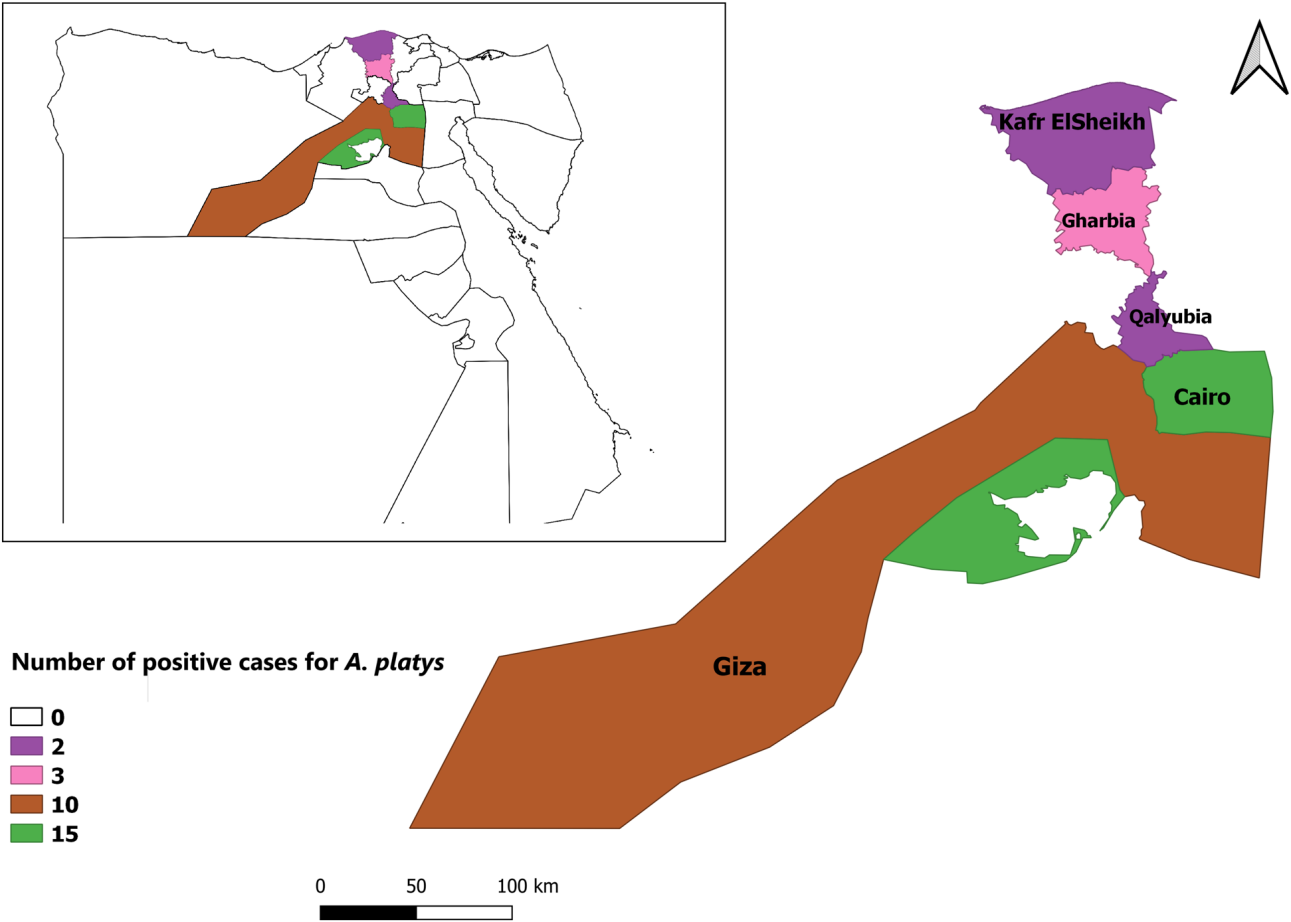
Map of Egypt indicating the studied governorates. Map was generated using QGIS software ver. 3.18.3 (https://qgis.org/en/site)

The study was carried out between December 2019 and November 2020. Blood samples were collected from 500 privately owned dogs of different breeds (i.e., German shepherd, Rottweiler and pit bull). Upon physical inspection, some dogs were apparently healthy, whereas others showed clinical signs suggestive of vector-borne pathogens (e.g., fever, lethargy and bleeding disorder). Sampling was done in the presence of a veterinarian and with the verbal consent of the owners. Blood samples were collected aseptically from the cephalic vein into EDTA tubes and kept at −20 °C until further processing. Data about sex, age, breed, tick infestation, anti-tick treatment and veterinary care were recorded and analyzed as possible risk factors.

For molecular analysis, genomic DNA was extracted from 200 µl whole blood sample of each dog using the QIAamp DNA Mini Kit^®^ (Qiagen, Valencia, USA) following the manufacturer’s instructions. A conventional polymerase chain reaction (PCR) assay targeting the *16S* rRNA gene was performed as previously described [24], using the primers PLATYS (GATTTTTGTCGTAGCTTGCTATG) and EHR16SR (TAGCACTCATCGTTTAC AGC), which produce an amplicon of 678 base pairs (bp). PCR was performed in a 25 µl volume, containing 1 µl of each primer (20 pmol/μl), 12.5 µl of DreamTaq Green PCR Master Mix (2×) (Thermo Scientific, Germany), 5.5 µl nuclease-free water and a 5 µl aliquot of isolated DNA. The thermal profile of the PCR was as follow: 95 °C for 5 min followed by 40 cycles of denaturation at 95 °C for 30 s, annealing at 55 °C for 30 s and extension at 72 °C for 90 s, and final extension of 72 °C for 5 min. The amplification products were visualized on a 2% agarose gel with ethidium bromide under UV light.

The PCR product of a positive sample was purified using the QIAquick PCR Purification Kit (QIAGEN, Valencia, CA, USA) and sequenced using the same primers as the conventional PCR assay. The sequencing was performed in a 3500 Genetic Analyzer (Applied Biosystems, USA) using the BigDye™ Terminator v3.1 Cycle Sequencing Kit (Applied Biosystems, USA) according to manufacturer’s protocol. The sequences obtained were assembled and edited using the BioEdit program and deposited in GenBank under accession number LC632659. The obtained sequence was aligned with other *Anaplasmataceae* *16S* rRNA gene sequences retrieved from GenBank using CLUSTAL W (http://www.clustalw.genome.jp). A phylogenetic tree was constructed using the neighbor-joining tree method with 1000 bootstrap replicates based on the Kimura 2-parameter model for nucleotide sequences using MEGA7 [16].

The data were analyzed using SPSS software (version 24.0, IBM Corp., Armonk, NY USA). Univariate and multivariate logistic regression analyses were used to evaluate the potential risk factors for *A. platys* infection. The odds ratios (OR), *P*-values (≤ 0.05) and 95% confidence intervals (95% CI) were evaluated to determine the strength of association between variables. A *P*-value ≤ 0.05 was considered significant. Analyzed risk factors included locality (Cairo, Giza, Qalyubia, Gharbia and Kafr El Sheikh), sex, age group (< 2, 2–5, and > 5 years), breed (German shepherd, Rottweiler and pit bull), tick infestation, anti-tick treatment and veterinary care.

A total of 500 blood samples were collected from domestic dogs and examined using conventional PCR targeting *16S* rRNA gene. The overall prevalence of *A. platys* in dogs was 6.4% (95% CI: 4.5–9%), ranging from 3.3 to 9.1% according to governorates; the highest prevalence rate was reported in Giza (9.1%, 95% CI: 4.6–16.4%). Nonetheless, there was no significant variation between different governorates (χ^2^ = 2.604, *df* = 4, *P* = 0.6), as shown in Table 1.

**Table 1:** Risk factors associated with prevalence of *Anaplasma platys* in domestic dogs, Egypt

Variable	*n*	No. of positive	Prevalence	95% CI	Statistics
Location					
Cairo	230	15	6.5	3.8–10.7	χ^2^ = 2.605, *df* = 4, *P* = 0.626
Giza	110	10	9.1	4.6–16.4	
Qalyubia	60	2	3.3	0.5–12.5	
Gharbia	60	3	5	1.3–14.8	
Kafr El Sheikh	40	2	5	0.8–18.2	
Sex					
Male	230	8	3.5	1.6–6.9	χ^2^ = 6.070, *df* = 1, *P* = 0.014*
Female	270	24	8.8	5.9–13.1	
Age					
≤ 2	160	8	5	2.3–9.9	χ^2^ = 0.884, *df* = 2, *P* = 0.642
2–5 year	260	19	7.3	4.5–11.3	
> 5 year	80	5	6.3	2.3–14.6	
Breed					
German shepherd	260	23	8.8	5.8–13.2	χ^2^ = 6.396, *df* = 2, *P* = 0.041*
Rottweiler	110	6	5.4	2.2–11.9	
Pit bull	130	3	2.3	0.6–7.1	
Tick infestation					
Yes	140	20	14.3	9.2–21.4	χ^2^ = 20.185, *df* = 1, *P* < 0.0001
No	360	12	3.3	1.8–5.9	
Anti-tick treatment					
Yes	370	12	3.2	1.8–5.5	χ^2^ = 23.673, *df* = 1, *P* < 0.0001*
No	130	20	15.4	9.9–23	
Veterinary care					
Yes	370	10	2.7	1.4–5.1	χ^2^ = 32.474, *df* = 1, *P* < 0.0001*
No	130	22	9.2	5.1–15.9	
Total	500	32	6.4	4.5–9	

*The result is statistically significant at *P* < 0.05

In the univariate analysis, the prevalence of *A. platys* was higher in female dogs and German shepherd breed (Table 1). The risk of infection with *A. platys* was significantly associated with lack of veterinary care, tick infestation and lack of anti-tick treatment (Table 1). In the multivariate logistic regression, *A. platys* infection was significantly associated with female sex, German shepherd breed, tick infestation, lack of veterinary care and lack of anti-tick treatment (Table 2).

**Table 2:** Multivariate logistic regression analysis of risk factors associated with *A. platys* infection in dogs, Egypt

Variable		*B*	SE	OR	95% CI	*P*-value
Sex	Female	1.574	0.399	4.8	2.2–10.5	< 0.0001*
Breed	German shepherd	1.413	0.624	4.1	1.2–13.9	0.02
	Rottweiler	0.893	0.132	2.4	0.6–10	0.2
Presence of ticks	Yes	1.576	0.38	4.8	2.3–10.2	< 0.0001*
Anti-tick treatment	No	1.805	0.375	6.1	2.9–12.6	< 0.0001*
Veterinary care	No	1.992	0.397	7.3	3.4–15.9	< 0.0001*

*B* logistic regression coefficient, *SE* standard error, *OD* odds ratio, *CI* confidence interval

*The result is statistically significant at *P* < 0.05

The obtained *16S* rRNA gene sequence showed nucleotide identity > 99% with other *A. platys* sequences from GenBank (MZ068099, MT053461, MT044313, MT044313) using BLAST. Phylogenetic analysis was performed using a 423 bp fragment of the *A. platys 16S* rRNA gene generated herein along with 21 *A. platys* sequences available in GenBank. The phylogenetic tree demonstrated that the sequence obtained herein is closely related to a sequence from Egypt (GenBank: MZ068099) and formed a cluster with other sequences from South Africa and Uruguay (Fig. 2). Nonetheless, additional data from a larger number of sequences are necessary for understanding the clinical-epidemiological significance (if any) of this phylogenetic finding.

**Fig. 2:**
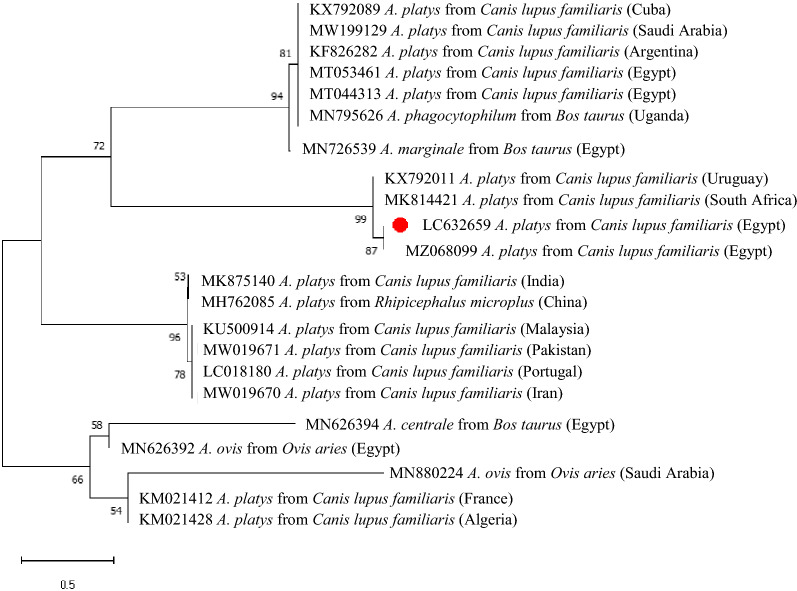
Phylogenetic tree of *A. platys* sequence identified in the current study based on *16S* rRNA gene. The analyses were performed using neighbor-joining tree method with 1000 bootstrap replicates based on Kimura 2-parameter model using MEGA7 [16]

Overall, the prevalence of *A. platys* in dogs in Egypt found herein (i.e., 6.4%) is similar to that reported in Argentina [17], and slightly higher than that reported in some studies in Italy [18], Croatia [19] and Mexico [20]. On the other hand, the prevalence found herein is lower than that reported in other studies conducted in Paraguay [30], Brazil [21], French Guiana [22] and Chile [23]. In Africa and Asia, previous studies were performed in different countries such as Algeria [12], Nigeria [24], South Africa [25], Malaysia [26], Iran [27] and Turkey [28], where the reported prevalence of *A. platys* infection ranged between 4.4 and 13.3%. These differences in the prevalence of *A. platys* infection may be attributed to local risk factors including climate, vector density, socioeconomic factors and lack of anti-tick preventatives, but also to methodological factors.

In this study, older dogs were more likely to be positive for *A. platys* than juvenile dogs, as reported in previous study in Egypt [14, 29]. This may be related to an increased risk of exposure during the dog’s life. Interestingly, no breed, age, or sex predisposition has been described for *A. platys* infection in dogs in Europe [30].

The positivity to *A. platys* was significantly higher in females than in males, as opposed to previous studies from Egypt [14, 31]. This may be related to higher tick exposure in females as compared to males in the area investigated in the present study. Concerning the breed, German shepherd dogs were more likely to be infected than other breeds, as reported previously in Egypt [14]. Again, no breed predisposition [30], or even slightly lower risk in purebred dogs [31], has been reported previously, which suggests that this apparent breed predisposition in German shepherd dogs in Egypt may be related to local factors that increase tick exposure in these dogs. Further studies on breed predisposition for *A. platys* infection in dogs are advocated. As expected, the absence of veterinary care and treatment against ticks were significant risk factors for *A. platys* infection, as reported previously [14, 32].

The sequencing of the partial *16S* rRNA gene showed high sequence identity with another *A. platys* isolate (GenBank: MZ068099) from Egypt. Furthermore, the phylogenetic analysis of the obtained *A. platys* strain clustered together with other reference strains of *A. platys* from Uruguay, South Africa and Egypt. The molecular identification of *A. platys* in some governorates of northern Egypt highlights the need for notification of veterinarians, dog owners and public health authorities to prevent the spread of vector-borne infections among dogs.

In conclusion, our study confirms the presence of *A. platys* in dogs from Egypt. The prevalence of disease was higher in females, particularly German shepherd dogs. In addition, absence of veterinary care, lack of anti-tick treatment and high tick infestation were identified as risk factors for *A. platys* infection in these dogs. The phylogenetic analysis confirmed the sequence identified herein with a previous Egyptian strain and other *A. platys* in GenBank.

## Data Availability

All data generated or analyzed during this study are included in this published article and its additional files.

## Abbreviations

*Anaplasma platys*: *A. platys*
UV: Ultraviolet
OR: Odds ratio
CI: Confidence interval
s.l.: Sensu lato

## References

[1] Sanogo Y, Inokuma H, Parola P, Brouqui P, Davoust B, Camicas J (2003). First evidence of *Anaplasma platys* in *Rhipicephalus sanguineus* (Acari: Ixodida) collected from dogs in Africa. Onderstepoort J Vet Res.

[2] Ramos RAN, Latrofa MS, Giannelli A, Lacasella V, Campbell BE, Dantas-Torres F (2014). Detection of *Anaplasma platys* in dogs and *Rhipicephalus sanguineus* group ticks by a quantitative real-time PCR. Vet Parasitol.

[3] Hegarty BC, Qurollo BA, Thomas B, Park K, Chandrashekar R, Beall MJ (2015). Serological and molecular analysis of feline vector-borne anaplasmosis and ehrlichiosis using species-specific peptides and PCR. Parasit Vectors.

[4] Pereira A, Parreira R, Nunes M, Casadinho A, Vieira ML, Campino L (2016). Molecular detection of tick-borne bacteria and protozoa in cervids and wild boars from Portugal. Parasit Vectors.

[5] Iatta R, Sazmand A, Nguyen V-L, Nemati F, Ayaz MM, Bahiraei Z, et al. Vector-borne pathogens in dogs of different regions of Iran and Pakistan. Parasitol Res. (in press). 10.1007/s00436-020-06992-x PMC8599219 33506332

[6] Cardoso L, Tuna J, Vieira L, Yisaschar-Mekuzas Y, Baneth G (2010). Molecular detection of *Anaplasma platys* and *Ehrlichia canis* in dogs from the North of Portugal. Vet J.

[7] Dyachenko V, Pantchev N, Balzer H-J, Meyersen A, Straubinger RK (2012). First case of *Anaplasma platys* infection in a dog from Croatia. Parasit Vectors.

[8] Rojas A, Rojas D, Montenegro V, Gutiérrez R, Yasur-Landau D, Baneth G (2014). Vector-borne pathogens in dogs from Costa Rica: first molecular description of *Babesia vogeli* and *Hepatozoon canis* infections with a high prevalence of monocytic ehrlichiosis and the manifestations of co-infection. Vet Parasitol.

[9] Santamaria A, Calzada JE, Saldana A, Yabsley MJ, Gottdenker NL (2014). Molecular diagnosis and species identification of *Ehrlichia* and *Anaplasma* infections in dogs from Panama, Central America. Vector Borne Zoonotic Dis.

[10] Nguyen V-L, Colella V, Greco G, Fang F, Nurcahyo W, Hadi UK (2020). Molecular detection of pathogens in ticks and fleas collected from companion dogs and cats in East and Southeast Asia. Parasit Vectors.

[11] Sarih MH, M’Ghirbi Y, Bouattour A, Gern L, Baranton G, Postic D (2005). Detection and identification of *Ehrlichia* spp. in ticks collected in Tunisia and Morocco. J Clin Microbiol.

[12] Dahmani M, Loudahi A, Mediannikov O, Fenollar F, Raoult D, Davoust B (2015). Molecular detection of *Anaplasma platys* and *Ehrlichia canis* in dogs from Kabylie. Algeria Ticks Tick Borne Dis.

[13] Seng P, Sarih M, Socolovschi C, Boudebouch N, Hassar M, Parola P (2009). Detection of Anaplasmataceae in ticks collected in Morocco. Clin Microbiol Infect.

[14] Selim A, Alanazi AD, Sazmand A, Otranto D (2021). Seroprevalence and associated risk factors for vector-borne pathogens in dogs from Egypt. Parasit Vectors.

[15] Nasr A, El Hariri M, Ghafar MW (2020). Detection of *Anaplasma platys* and *Ehrlichia canis* in *Rhipicephalus sanguineus* ticks attached to dogs from Egypt; a public health concern. Vet Med J Giza.

[16] Kumar S, Stecher G, Tamura K (2016). MEGA7: molecular evolutionary genetics analysis version 7.0 for bigger datasets. Mol Biol Evol.

[17] Cicuttin GL, De Salvo MN, Dohmen FEG (2016). Molecular characterization of *Ehrlichia canis* infecting dogs, Buenos Aires. Ticks Tick Borne Dis.

[18] Trotta M, Fogliazza A, Furlanello T, Solano-Gallego L (2009). A molecular and serological study of exposure to tick-borne pathogens in sick dogs from Italy. Clin Microbiol Infect.

[19] Huber D, Reil I, Duvnjak S, Jurković D, Lukačević D, Pilat M (2017). Molecular detection of *Anaplasma platys*, *Anaplasma phagocytophilum* and *Wolbachia* sp. but not *Ehrlichia canis* in Croatian dogs. Parasitol Res.

[20] Almazán C, González-Álvarez VH, de Mera IGF, Cabezas-Cruz A, Rodríguez-Martínez R, de la Fuente J (2016). Molecular identification and characterization of *Anaplasma platys* and *Ehrlichia canis* in dogs in Mexico. Ticks Tick Borne Dis.

[21] Costa-Júnior L, Rembeck K, Passos L, Ribeiro M (2013). Factors associated with epidemiology of *Anaplasma platys* in dogs in rural and urban areas of Minas Gerais State, Brazil. Prev Vet Med.

[22] Dahmani M, Marié J-L, Mediannikov O, Raoult D, Davoust B (2015). First identification of *Anaplasma platys* in the blood of dogs from French Guiana. Vector Borne Zoonotic Dis.

[23] Abarca K, López J, Perret C, Guerrero J, Godoy P, Veloz A (2007). *Anaplasma platys* in dogs. Chile Emerg Infect Dis.

[24] Daramola OO, Takeet MI, Oyewusi IK, Oyekunle MA, Talabi AO (2018). Detection and molecular characterisation of *Ehrlichia canis* in naturally infected dogs in South West Nigeria. Acta Vet Hung.

[25] Matjila PT, Leisewitz AL, Jongejan F, Penzhorn BL (2008). Molecular detection of tick-borne protozoal and ehrlichial infections in domestic dogs in South Africa. Vet Parasitol.

[26] Mokhtar A, Lim S, Tay S (2013). Research note molecular detection of *Anaplasma platys* and *Babesia gibsoni* in dogs in Malaysia. Trop Biomed.

[27] Maazi N, Malmasi A, Shayan P, Nassiri SM, Salehi TZ, Fard MS (2014). Molecular and serological detection of *Ehrlichia canis* in naturally exposed dogs in Iran: an analysis on associated risk factors. Rev Bras Parasitol Vet.

[28] Cetinkaya H, Matur E, Akyazi I, Ekiz EE, Aydin L, Toparlak M (2016). Serological and molecular investigation of *Ehrlichia* spp. and *Anaplasma* spp. in ticks and blood of dogs, in the Thrace Region of Turkey. Ticks tick Borne Dis..

[29] Guedes PEB, Oliveira TNdA, Carvalho FS, Carlos RSA, Albuquerque GR, Munhoz AD (2015). Canine ehrlichiosis: prevalence and epidemiology in northeast Brazil. Rev Bras Parasitol Vet.

[30] Sainz Á, Roura X, Miró G, Estrada-Peña A, Kohn B, Harrus S (2015). Guideline for veterinary practitioners on canine ehrlichiosis and anaplasmosis in Europe. Parasit Vectors.

[31] Pesapane R, Foley J, Thomas R, Castro LR (2019). Molecular detection and characterization of *Anaplasma platys* and *Ehrlichia canis* in dogs from northern Colombia. Vet Microbiol.

[32] Pérez-Macchi S, Pedrozo R, Bittencourt P, Müller A (2019). Prevalence, molecular characterization and risk factor analysis of *Ehrlichia canis* and *Anaplasma platys* in domestic dogs from Paraguay. Comp Immunol Microbiol Infect Dis.

